# Extracts of *Magnolia* Species-Induced Prevention of Diabetic Complications: A Brief Review

**DOI:** 10.3390/ijms17101629

**Published:** 2016-09-24

**Authors:** Xuezhong Zhao, Fengsheng Li, Wanqing Sun, Ling Gao, Ki Soo Kim, Kyoung Tae Kim, Lu Cai, Zhiguo Zhang, Yang Zheng

**Affiliations:** 1 Departments of Cardiology at the First Hospital of Jilin University, Changchun 130021, China; zhxzhzxz@163.com; 2 Kosair Children Hospital Research Institute, Department of Pediatrics, University of Louisville, Louisville, KY 40202, USA; lifs0624@163.com (F.L.); L0cai001@louisville.edu (L.C.); 3 General Hospital of the PLA Rocket Force, Beijing 100088, China; 4 National Center for Cardiovascular Diseases China, Fuwai Hospital, Chinese Academy of Medical Sciences and Peking Union Medical College, Beijing 100037, China; sunwq1986@hotmail.com; 5 Key Laboratory of Radiological Protection and Nuclear Emergency, National Institute for Radiological Protection, China Centers for Disease Control, Beijing 100088, China; gaolingxinxin@126.com; 6 The Bioland Biotec Co., Ltd., Zhangjiang Modern Medical Device Park, Pudong, Shanghai 201203, China; cuttyfamily@hotmail.com (K.S.K.); kaekkt@hotmail.com (K.T.K.)

**Keywords:** *Magnolia*, diabetic complications, oxidative stress

## Abstract

Diabetic complications are the major cause of mortality for the patients with diabetes. Oxidative stress and inflammation have been recognized as important contributors for the development of many diabetic complications, such as diabetic nephropathy, hepatopathy, cardiomyopathy, and other cardiovascular diseases. Several studies have established the anti-inflammatory and oxidative roles of bioactive constituents in *Magnolia* bark, which has been widely used in the traditional herbal medicines in Chinese society. These findings have attracted various scientists to investigate the effect of bioactive constituents in *Magnolia* bark on diabetic complications. The aim of this review is to present a systematic overview of bioactive constituents in *Magnolia* bark that induce the prevention of obesity, hyperglycemia, hyperlipidemia, and diabetic complications, including cardiovascular, liver, and kidney.

## 1. Introduction

Diabetes mellitus (DM) is a chronic metabolic disease defined by elevated glycemic markers, and is associated with disrupted insulin secretion, insulin resistance, and lipid metabolic disorder. DM has become a major public health concern in the world today, creating a lot of therapeutic problems. Untreated diabetes leads to a wide array of complications, including heart failure, nonalcoholic fatty liver disease, renal failure, and macrovascular disturbances. New drugs are still being sought to treat diabetic patients. Many natural plants are known to have promising antidiabetic properties [1,2,3,4]. For example, bioactive constituents of *Magnolia* attenuated hyperglycemia [5], prevented cardiac pathogenesis [6] and live damage [7] and histologic renal damage [8] in diabetes and obesity.

**Figure 1:**
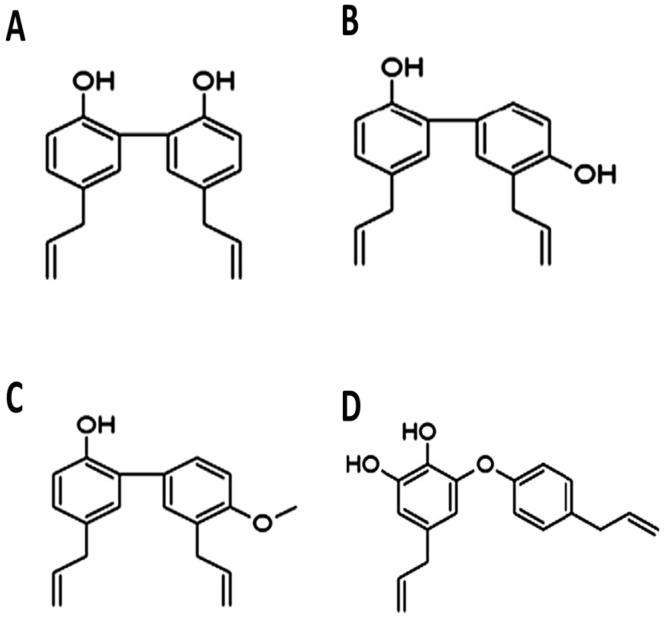
Chemical structures of (**A**) magnolol; (**B**) honokiol; (**C**) 4-*O*-methylhonokiol; and (**D**) obovatol.

*Magnolia* is a Chinese herbal medicine which has been used in traditional medicine for a long time in China. The flower and bark of *Magnolia* have been widely used as traditional herbal remedy for various disorders such as headache, fever, anxiety, diarrhea, stroke, and asthma. The genus *Magnolia* has been reported to exert various biological effects, including anticarcinogenicity [9], anti-inflammatory effects [10], antioxidative stress [11], and antianxiety [12]. In the cardiovascular system, it showed vascular relaxation, antiatherosclerosis, and antiplatelet effects. Honokiol, magnolol, 4-*O*-methylhonokiol (MH), and obovatol are considered major bioactive constituents of *Magnolia* stem bark (Figure 1) [13].

Previous phytochemical investigations have reported that this species contains several secondary metabolites such as lignans, neolignans, sesquiterpenes, and essential oils, which express various biological activities. Recent studies have suggested that the constituents of *Magnolia* ameliorated characters of obesity and diabetes, such as hyperglycemia, hyperlipidemia and complications of diabetes (Table 1). This review aims to provide mechanistic insights by highlighting the relationship between constituents of *Magnolia* genus and diabetes, and their contribution in the prevention of complications.

## 2. The Effect of *Magnolia* Genus on Blood Glucose

**Figure 2:**
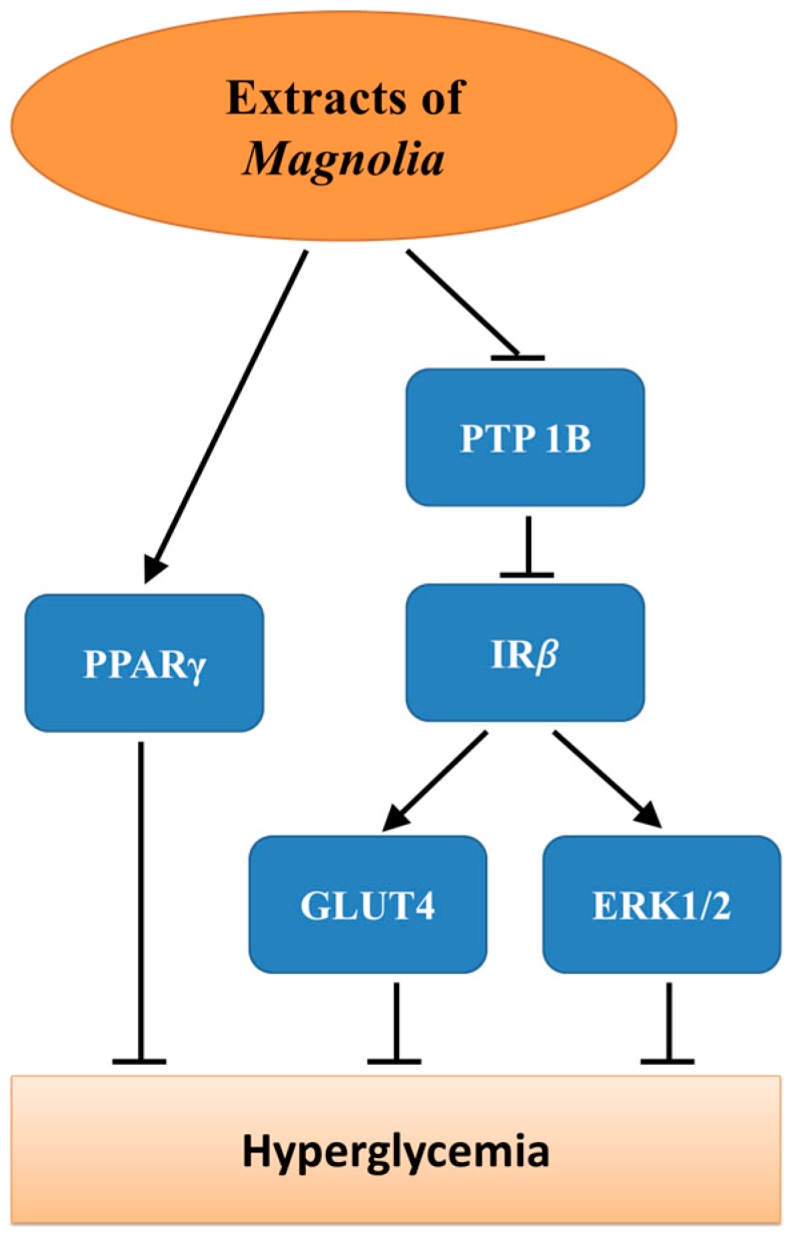
The underlying mechanism through which bioactive constituents of *Magnolia* bark prevent hyperglycemia of diabetes. PTP1B: protein tyrosine phosphatases (PTPs) 1B; IR*β*: insulin receptor *β*-subunit; PPARγ: peroxisome proliferator-activated receptor gamma; GLUT4: glucose transporter 4; ERK1/2: extracellular signal-regulated kinases.

Glycemic control is considered to be the most effective approach for the prevention of diabetic complications. Several studies have reported that most of the major bioactive constituents of *Magnolia* bark contribute to glycemic control (Figure 2) [14,15]. An in vitro study showed that honokiol and magnolol could promote the glucose uptake of adipocytes derived from human or murine in a concentration-dependent manner through insulin signaling pathway [16]. These findings were in line with the results of Choi’s study [15] and Atanasov’s study [14], in which magnolol and honokiol were reported to enhance basal glucose uptake of mouse preadipocytes 3T3-L1 cells, respectively.

More importantly, in vivo studies using diabetic animal models have confirmed that bioactive constituents of *Magnolia* bark were promising hypoglycemic bioactivity. Using a type 2 diabetes (T2DM) mouse model established by high-fat diet (HFD) combining with streptozotocin (STZ) injection, Sun et al. [17] demonstrated that oral gavage of honokiol at dose of 200 mg/kg once per day for 8 weeks significantly decreased the blood glucose levels. Sun et al. [5] also investigated the effect of *Magnolia officinalis* extract on blood glucose level of db/db mice which have been recognized as a model of T2DM. The authors found that *Magnolia* extracts (ME) treatment once a day at dose of 0.5 g/kg for 4 weeks attenuates hyperglycemia in db/db mice. Another study reported that treatment with honokiol at a lower dose (100 mg/kg once per day for 5 weeks) could prevent hyperglycemia of KKAy mice [14]. Actually, a much lower dose of honokiol or magnolol (17 mg/kg once per day for 16 weeks) could effectively ameliorate the insulin resistance of HFD fed mice, although fasting blood glucose and plasma insulin levels were not improved [18]. These studies indicated that high dose (200 mg/kg) and low dose (100 mg/kg) honokiol could decrease the blood glucose levels in diabetic mice. However, much lower dose (17 mg/kg) honokiol for long time (16 weeks) did not improve hypoglycemia and insulin levels. The reason for the different doses of ME and constituents used in the different studies probably is that methods for purifying and isolating ME were different, which is due to different bioavailability of the bioactive compounds after absorption.

The glycemic control mechanism of bioactive constituents of *Magnolia* bark has been proven to be associated with the enhancement of insulin-signaling pathway. Sun et al. [5] demonstrated that in vitro treatment with *Magnolia officinalis* extracts enhanced the phosphorylation of insulin receptor β-subunit (IRβ) in response to insulin stimulation in 3T3-L1 adipocytes and C2C12 myotubes by suppressing the activity of protein tyrosine phosphatases 1B, which finally resulted in enhanced insulin-stimulated glucose transporter 4 (GLUT4) translocation and extracellular signal-regulated kinases phosphorylation. Furthermore, it has been reported that honokiol can act as an agonist of peroxisome proliferator-activated receptor gamma, which plays an important role in regulation of glucose homeostasis [14].

## 3. The Effect of *Magnolia* Genus on Hyperlipidemia and Obesity

Dysregulation of fatty acid metabolism, mainly caused by insulin resistance among multiple tissues, is a primarily pathological change in the patients with T2DM, and characterized by elevated levels of plasma free fatty acid (FFA), and increased triglyceride (TG) content in in various tissues, including skeletal muscle, cardiac muscle, and liver [19,20,21,22]. Dysregulation of fatty acid metabolism has been considered to play a key role in the development of diabetic complications [23,24,25]. Moreover, it has been reported that high circulating FFA levels in turn aggravated the development of diabetes by prompting beta-cell dysfunction and insulin resistance [26].

**Figure 3:**
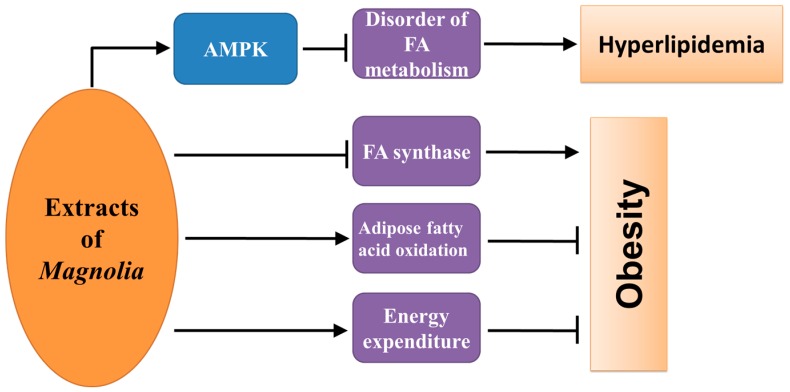
The underlying mechanism through which bioactive constituents of *Magnolia* bark prevent obesity and hyperlipidemia of diabetes. AMPK: AMP-activated protein kinase; FA: fatty acid.

The major bioactive constituents of *Magnolia* bark can ameliorate dysregulation of fatty acid metabolism caused by diabetes (Figure 3). Honokiol attenuated intracellular fat over loading and TG accumulation in FFA-exposed HepG2 cells through activating liver kinase B1/AMP-activated protein kinase (AMPK) signaling pathway [27]. In mice, the increase in hepatic TG and fat accumulation caused by HFD was ameliorated by honokiol [27] or MH treatments [28]. Moreover, the treatment with MH also decreased HFD-increased plasma TG and cholesterol levels [28]. In addition, the weight of visceral white adipose tissue (WAT) in mice fed with HFD also decreased after long-term supplementation of honokiol or magnolol due to honokiol- or magnolol-induced upregulation of energy expenditure and adipose fatty acid oxidation, and downregulation of fatty acid synthase activity and expression of fatty acid synthesis-related gene [18]. The weight gain was also suppressed by honokiol treatment in a study using diabetic KKAy mice [14], which was in correlation with the findings in one of the studies we conducted, where MH, a bioactive constituent of *Magnolia* extract BL153, attenuated HFD-induced obesity [6].

## 4. The Effect of *Magnolia* Genus on Cardiovascular System of Diabetic Subjects

Diabetic heart disease is one of major complications of diabetes, and includes coronary heart disease (CHD), heart failure, and diabetic cardiomyopathy (DCM), among which DCM is one of major cardiac complications of diabetes. It is presented by coronary artery disease- and hypertension-independent structural and functional changes of heart, including cardiac hypertrophy and compromised systolic and diastolic function [29]. Although the underlying mechanisms for the pathogenesis of DCM are not fully elucidated, diabetes-caused oxidative stress, inflammation and lipid accumulation are recognized as crucial contributors for the development of DCM [30,31,32].

**Figure 4:**
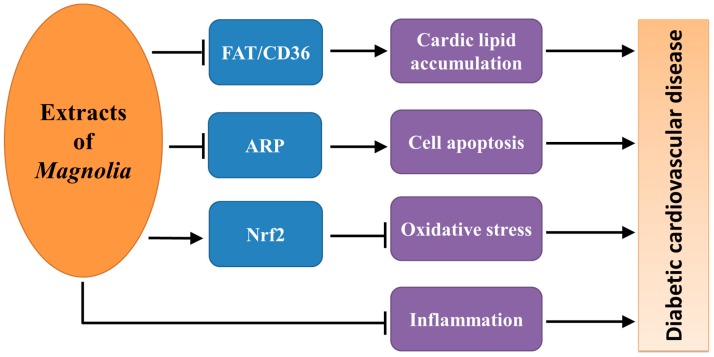
The underlying mechanism through which bioactive constituents of *Magnolia* bark prevent diabetic cardiovascular disease. FAT: fatty acid translocase; ARP, apoptosis related proteins, including Fas ligand, caspase 8, and caspase 9; Nrf2: nuclear factor erythroid-derived 2-like 2.

Drugs that treat heart failure are beneficial in DCM, however, the specifically preventive and therapeutic approach for DCM is still unavailable up to now [32]. In one of our recent studies, we have showed that *Magnolia* extract (BL153) treatment, at dose of 10 mg/kg body weight daily, slightly attenuated a mild cardiac hypertrophy and dysfunction of mice induced by HFD feeding [4]. Furthermore, we showed that BL153 treatment could attenuate cardiac lipid accumulation, oxidative damage, inflammation, and cell death in the heart of mice with HFD [4], highlighting the potential underlying mechanism for BL153 against DCM. These exciting findings encouraged the authors to further study the effect of BL153 bioactive constituent on HFD-induced cardiac pathogenesis. As expected, treatment with MH, a bioactive constituent of BL153, significantly decreased HFD-induced structural change of heart, which was concluding by the fact that HFD-induced increases in heart weight and ventricular wall thickness were significantly attenuated by MH treatment [6]. This study further confirmed that MH treatment resulted in the activation of Akt2 and nuclear factor erythroid-derived 2-like 2 (Nrf2) signaling which reduced HFD-induced impairment of cardiac insulin signaling and decreased oxidative stress and damage, respectively [6]. In addition, MH reduced HFD-induced cardiac lipid accumulation by suppressing cardiac fatty acid translocase/CD36 protein expression. CHD is the leading cause of mortality in diabetic patients and correlated with diabetes-accelerated formation and/or progression of atherosclerotic lesions [33,34]. Although there is no report from literature relevant to the direct therapeutic effect of *Magnolia* bark on CHD, a study using HFD feeding rabbit model showed that *Magnolia officinalis* treatment suppressed arterial atherosclerosis progression through suppression of oxidative stress and apoptosis-related gene expression, two major contributors for the progression of atherosclerosis, which indicated that *Magnolia officinalis* treatment might be beneficial for the prevention of CHD [35]. Therefore, the major bioactive constituents of *Magnolia* bark have contributed to prevent cardiovascular disease (Figure 4), although the mechanism is still not clear.

## 5. The Effect of *Magnolia* Genus on the Kidney of Diabetic Subjects

It has been recognized that diabetes mellitus is a major contributor for chronic kidney disease worldwide, and diabetic nephropathy (DN), characterized by the accumulation of extracellular matrix protein in the glomerular mesangium and tubulointerstitium, is one of the major causes of death in patients with diabetes [36]. The pathogenesis of DN is multifactorial, and many factors have been reported to be associated with DN, including the hyperglycemia-caused accumulation of advanced glycation end products (AGEs), oxidative stress, and inflammation [37,38]. Therefore, AGEs inhibitor and anti-inflammatory or antioxidant drugs have been proposed as promising agents for the treatment of DN [38,39].

**Figure 5:**
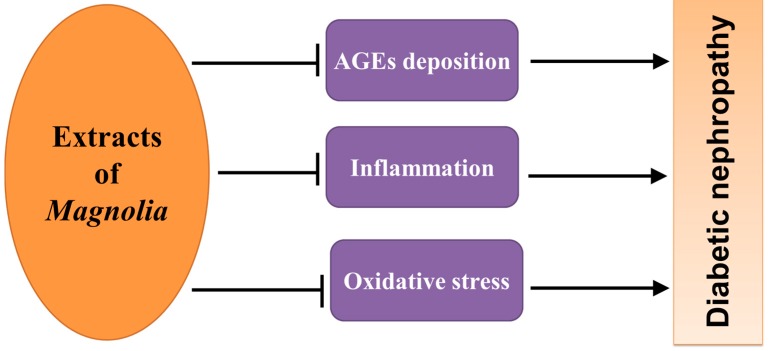
The underlying mechanism through which bioactive constituents of *Magnolia* bark prevent diabetic nephropathy.

The protective effect of bioactive constituents in *Magnolia* bark on DN has been investigated in many studies using diabetic or HFD feeding animal model (Figure 5). Using Zucker diabetic fatty rats, Kim et al. [8] firstly reported that administration of KIOM-79, extracted from *Magnolia* cortex, at dose of 50 mg/kg daily for 13 weeks could ameliorate albuminuria, histologic renal damage, glomerulosclerosis, tubular degeneration, collagen deposition, and podocyte apoptosis. Another study found that the in vitro AGE-protein cross-linking and in vivo accumulation of AGEs in renal cortex of diabetic rat was attenuated by KIOM-79 treatment, indicating that the protective effect of KIOM-79 on DN was involved in attenuation of AGEs deposition in the glomeruli [8].

The above-discussed renal protection by bioactive constituents in *Magnolia* bark from diabetes was also confirmed in other types of diabetes. Using diabetic rats induced by STZ injection, magnoline was also found to be renoprotective from diabetes-caused damage, shown by lower albuminuria and serum creatinine, when diabetic rats were given magnoline at dose of 0.5 mg/kg daily or 2 mg/kg daily [40]. Our group previously explored the protective effect of BL153 on kidney damage induced by HFD, and found that BL153 treatment significantly decreased HFD-induced renal dysfunction and structure changes [41]. More importantly, the elevated expressions of inflammation markers (tumor necrosis factor-α and plasminogen activator inhibitor-1) and oxidative stress markers (3-nitrotyrosine and 4-hydroxy-2-nonenal) in kidney of mice with HFD were attenuated by BL153 treatment, revealing that renoprotective effect of BL153 might be mediated by its anti-inflammation and anti-oxidative stress action [41].

## 6. The Effect of *Magnolia* Genus on the Liver of Diabetic Subjects

Besides heart and kidney damage, diabetes also increases the risk of chronic liver injury [42,43,44,45], whose pathological features include excessive glycogen deposits in hepatocytes, hepatic steatosis, inflammatory cells infiltration in the lobule and portal area, and interstitial fibrotic proliferation [46]. Inflammation and oxidative stress, which can be attenuated by bioactive constituents of *Magnolia* bark as described above, play major roles during the development of liver injury secondary to diabetes [47,48,49].

**Figure 6:**
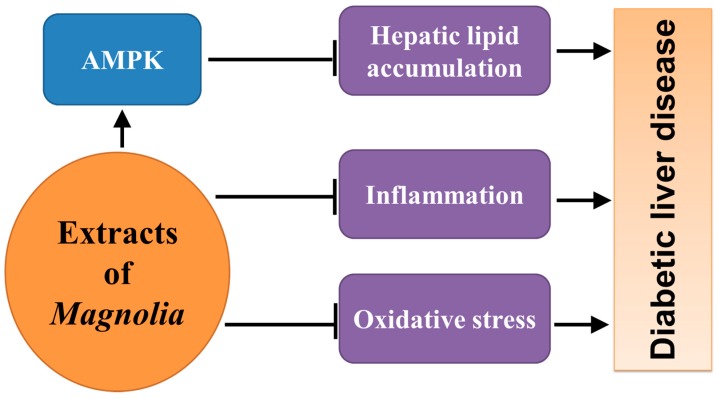
The underlying mechanism through which bioactive constituents of *Magnolia* bark prevent diabetic liver disease.

Using mice with HFD, our group firstly investigated the effect of *Magnolia* extract, BL153 on HFD-induced liver injury, and reported that BL153 treatment significantly suppressed HFD-induced hepatic fibrosis [50]. Further study showed that BL153 significantly inhibited HFD-induced hepatic lipid accumulation and oxidative stress and slightly prevented liver inflammation, which was the underlying mechanism for BL153 against HFD-induced hepatic fibrosis [50]. In a further study, Lee et al. [7] found that combination of honokiol and magnolol inhibited in vitro lipogenesis mediated by liver X receptor α, a nuclear receptor that regulates the metabolism of lipids, in hepatocytes through activation of AMPK, and ameliorated HFD-induced hepatic steatosis and liver dysfunction. The bioactive constituents of *Magnolia* bark therefore may be effective in treating diabetes-induced liver injury (Figure 6).

## 7. Conclusions

In the Chinese traditional herbal medicines, *Magnolia* bark has been used in the treatment of various diseases for many years, such as depression-related diseases and swallowing reflex in Parkinson's disease [51,52]. In vitro and in vivo studies disclosed that the therapeutic effect of *Magnolia* bark on several diseases partially depends on the anti-inflammatory and oxidative-stress role of bioactive constituents in *Magnolia* bark [10,11]. In recent years, there has been increasing attention to the effect of bioactive constituents in *Magnolia* bark on diabetic complications that have been considered due to the inflammation and oxidative stress induced by diabetic hyperglycemia and hyperlipidemia. Here we have presented an overview of these studies and found that in animal models of diabetes and HFD-induced obesity with and without diabetes, the bioactive constituents of *Magnolia* bark can improve hyperglycemia and ameliorate diabetic complications, including diabetes-caused dysregulation of fatty acid metabolism and damage to the heart, kidney, and liver (Table 1).

Magnolol, honokiol, and 4-*O*-methylhonokiol in the ME were used in most studies, however, other constituents maybe also have therapeutic effects. Mechanisms of antidiabetes are still unclear for *Magnolia* constituents. Therefore, ME are not used in antidiabetic clinical trial, though, clinical trials have demonstrated that ME may be important medicines for treating a variety of conditions such as menopause [53], anxiety [54], and gingivitis [55]. Now, it is necessary that we identify the bioactive constituents of ME, and delineate possible mechanisms. We hope that the brief review provides a foundation for further studies to assess mechanisms underlying the effects of ME, and clinical applications of these constituents.

Mechanistically these studies have linked the anti-inflammatory and antioxidative effect of *Magnolia* compounds to its efficiently preventive effects on the diabetic complications. These interesting studies strongly indicate that the bioactive constituents in *Magnolia* bark may be promising candidate agents for the treatment of diabetic complications in the future clinical research.

## Figures and Tables

**Table 1:** The effect of *Magnolia* components on obesity or diabetes complications.

Disease	Extracts	Models	Effect	Reference
Hyperglycemia	honokiol	Mice received HFD combining with STZ injection	Decrease in the blood glucose levels	[17]
	*Magnolia officinalis* extract	db/db mice	Attenuation of hyperglycemia	[5]
	honokiol	KKAy mice	Prevention of hyperglycemia	[18]
Hyperlipidemia and obesity	honokiol	Mice with HFD	Reduction of hepatic TG and fat accumulation	[27]
	4-*O*-methylhonokiol	Mice with HFD	Decrease in hepatic TG and fat accumulation	[28]
	honokiol or magnolol	Mice with HFD	Weight reduction of WAT	[18]
	honokiol	KKAy mice	Suppression of weight gain	[14]
	4-*O*-methylhonokiol	Mice with HFD	Suppression of weight gain	[6]
Diabetic heart disease	BL153	Mice with HFD	Attenuation of a mild cardiac hypertrophy and dysfunction	[4]
	4-*O*-methylhonokiol	Mice with HFD	Decrease in heart weight and ventricular wall thickness	[6]
	*Magnolia officinalis*	Mice with HFD	Suppression of arterial atherosclerosis progression	[35]
Diabetic kidney disease	KIOM-79	Zucker diabetic fatty rats	Amelioration of albuminuria, histologic renal damage, glomerulosclerosis, tubular degeneration, collagen deposition and podocyte apoptosis	[8]
	magnoline	Diabetic rats induced by STZ injection	Decreased albuminuria and serum creatinine	[40]
	BL153	Mice with HFD	Inhibition of renal dysfunction and structure changes	[41]
Diabetic liver disease	BL153	Mice with HFD	Suppression of hepatic fibrosis and hepatic lipid accumulation	[50]
	combination of honokiol and magnolol	Mice with HFD	Inhibition of hepatic steatosis and liver dysfunction	[7]

## Abbreviations

MH	4-*O*-methylhonokiol
T2DM	Type 2 Diabetes
HFD	High-fat diet
GLUT4	Glucose transporter 4
FFA	Free Fatty Acid
TG	Triglyceride
AMPK	AMP-activated protein kinase
WAT	White Adipose Tissue
CHD	Coronary Heart Disease
DCM	Diabetic Cardiomyopathy
Nrf2	Nuclear factor erythroid-derived 2-like 2
DN	Diabetic Nephropathy
AGEs	Advanced Glycation End products
